# Integrated Omics Analysis of Non-Small-Cell Lung Cancer Cells Harboring the EGFR C797S Mutation Reveals the Potential of AXL as a Novel Therapeutic Target in TKI-Resistant Lung Cancer

**DOI:** 10.3390/cancers13010111

**Published:** 2020-12-31

**Authors:** Tong-Hong Wang, Chih-Ching Wu, Kuo-Yen Huang, Yann-Lii Leu, Shuenn-Chen Yang, Ci-Ling Chen, Chi-Yuan Chen

**Affiliations:** 1 Graduate Institute of Health Industry Technology and Research Center for Chinese Herbal Medicine, College of Human Ecology, Chang Gung University of Science and Technology, Taoyuan 333, Taiwan; cellww@cgmh.org.tw; 2 Tissue Bank, Chang Gung Memorial Hospital at Linkou, Taoyuan 333, Taiwan; ylleu@mail.cgu.edu.tw; 3 Department of Medical Biotechnology and Laboratory Science, College of Medicine, Chang Gung University, Taoyuan 333, Taiwan; luckywu@mail.cgu.edu.tw; 4 Molecular Medicine Research Center, Chang Gung University, Taoyuan 333, Taiwan; 5 Department of Otolaryngology-Head&Neck Surgery, Chang Gung Memorial Hospital at Linkou, Taoyuan 333, Taiwan; 6 Institute of Biomedical Sciences, Academia Sinica, Taipei 115, Taiwan; kyhuang@ibms.sinica.edu.tw (K.-Y.H.); jjyang@ibms.sinica.edu.tw (S.-C.Y.); gling1994@ibms.sinica.edu.tw (C.-L.C.); 7 Department and Graduate Institute of Microbiology and Immunology, National Defense Medical Center, Taipei 11490, Taiwan; 8 Graduate Institute of Natural Products, Chang Gung University, Taoyuan 333, Taiwan

**Keywords:** NSCLC, EGFR C797S, EGFR T790M, TKI Resistance, AXL

## Abstract

**Simple Summary:**

In this study, we employed CRISPR/Cas9 editing technology to introduce the EGFR C797S mutation into an NSCLC cell line harboring EGFR L858R/T790M to establish a cellular model for the investigation of the resistance mechanism associated with the acquired C797S mutation and to explore strategies to battle this type of TKI resistance. Transcriptome and proteome analyses revealed that the differentially expressed genes/proteins in the cells harboring the EGFR C797S mutation are associated with elevated expression of AXL. Furthermore, we presented evidence that inhibition of AXL is effective in slowing the growth of NSCLC cells harboring EGFR C797S. Our findings suggest that AXL inhibition could be a second-line or a potential adjuvant treatment for NSCLC harboring the EGFR C797S mutation.

**Abstract:**

Oncogenic mutations of epidermal growth factor receptor (EGFR) are responsive to targeted tyrosine kinase inhibitor (TKI) treatment in non-small-cell lung cancer (NSCLC). However, NSCLC patients harboring activating EGFR mutations inevitably develop resistance to TKIs. The acquired EGFR C797S mutation is a known mechanism that confers resistance to third-generation EGFR TKIs such as AZD9291. In this work, we employed CRISPR/Cas9 genome-editing technology to knock-in the EGFR C797S mutation into an NSCLC cell line harboring EGFR L858R/T790M. The established cell model was used to investigate the biology and treatment strategy of acquired EGFR C797S mutations. Transcriptome and proteome analyses revealed that the differentially expressed genes/proteins in the cells harboring the EGFR C797S mutation are associated with a mesenchymal-like cell state with elevated expression of AXL receptor tyrosine kinase. Furthermore, we presented evidence that inhibition of AXL is effective in slowing the growth of NSCLC cells harboring EGFR C797S. Our findings suggest that AXL inhibition could be a second-line or a potential adjuvant treatment for NSCLC harboring the EGFR C797S mutation.

## 1. Introduction

Activating mutations of epidermal growth factor receptor (EGFR) are one of the most common oncogenic drivers in non-small cell lung cancer (NSCLC) and are detected in approximately 40% of East Asian patients and 15% of Caucasian patients [1]. These tumors have been termed oncogene addicted to reflect their dependence on the EGFR pathway and their susceptibility to survival inhibition by EGFR tyrosine kinase inhibitors (TKIs) [2]. Three generations of EGFR TKIs have been approved for the first-line treatment of NSCLC patients carrying activating EGFR mutations [2,3]. The first generation (gefitinib and erlotinib) and second generation (afatinib) of TKIs were designed to bind to the ATP binding pocket of the EGFR kinase domain [4]. These drugs are superior to chemotherapy in prolonging the progression-free survival (PFS) of NSCLC patients carrying common EGFR mutations, including exon 19 deletion (Del19) and exon 21 substitution (L858R). However, resistance to these TKIs developed in 60–80% of patients within 9–14 months after treatment [5,6,7]. The EGFR T790M substitution in exon 20, which inhibits the affinity of these TKIs by increasing steric hindrance, is the most common mechanism for acquired TKI resistance [8,9].

To overcome the resistance caused by T790M, third-generation TKIs that irreversibly interact with the cysteine 797 residue of EGFR and have more specificity for targeting T790M were developed, including AZD9291 (osimertinib), CO-1686 (rociletinib), EGF816, WZ4002, and ASP8273 [10,11]. Several third-generation TKIs have been tested in clinical trials, and AZD9291 was approved by the US Food and Drug Administration in 2017 to treat NSCLC patients with the T790M mutation. AZD9291 significantly improved PFS in EGFR T790M NSCLC patients who had disease progression on first-line TKIs [12]. AZD9291 has been assessed as a first-line treatment for EGFR activated-mutation NSCLC and has demonstrated a significant improvement in median PFS when compared to treatment with first generation TKIs [13]. Unfortunately, resistance to AZD9291 ultimately developed after a median PFS of 9.6 months [14].

Mechanisms involved in AZD92921 resistance are not fully understood and appear to be very heterogeneous. Resistance to AZD9291 treatment can be divided into EGFR-dependent and EGFR-independent mechanisms. The first resistance mechanism identified in patients was the tertiary EGFR mutation C797S, which directly targets the EGFR fixation site of AZD9291 [15]. Other EGFR mutations (e.g., EGFR L692V, G709L, or C797G) [16] and EGFR amplification have been described as alternative resistance mechanisms. EGFR-independent resistance mechanisms consist of activation of alternative pathways through different kinds of mutations (e.g., BRAF, KRAS, AKT, or PTEN) or gene amplifications (mainly EGFR, ERBB2, MET, KRAS, or PIK3CA) [3]. Moreover, cellular changes such as small-cell lung cancer (SCLC) transformation have been described as another resistance mechanism to third-generation EGFR TKIs [17].

While the mechanisms of acquired resistance to irreversible TKIs remain poorly understood, the acquired C797S mutation is a well-characterized mechanism of resistance. Patients with this class of resistance can be genotyped, and treatment based on molecular targeting of this mutated EGFR can be applied if available. Understanding the mechanisms of resistance and potential therapeutic strategies to acquired C797S mutation would be instrumental for the treatment of this class of patients. However, there is no good cellular model to dissect the mechanism of resistance to TKIs in EGFR-C797S-mutant lung cancer. In this study, we employed CRISPR/Cas9 (clustered regularly interspaced short palindromic repeats/CRISPR associated protein 9) editing technology to knock-in the EGFR C797S mutation into an NSCLC cell line harboring EGFR L858R/T790M to establish a cellular model for studying the biology and treatment strategy of acquired EGFR C797S mutations. We employed proteomics and transcriptome approaches to examine the genes that are differentially expressed in cells harboring the EGFR C797S mutation. Here, we present evidence that the differentially expressed genes in cells harboring the EGFR C797S mutation are associated with a mesenchymal-like cell state with elevated expression of AXL. Finally, an AXL-based treatment strategy to combat this class of TKI resistance was investigated.

## 2. Results

### 2.1. Knock-in of the EGFR C797S Mutation into H1975 Cells

To establish cellular models for the investigation of EGFR TKI resistance associated with the acquired C797S mutation in EGFR, we used CRISPR/Cas9 genome editing technology to knock-in the EGFR C797S mutation in NSCLC H1975 cells. Our overall study design was to employ the EGFR C797S knock-in cells to investigate the biological processes associated with the acquired EGFR C797S mutation by proteomics and transcriptome studies, and to explore the therapeutic strategies. We used an all-in-one CRISPR/Cas9 expression vector to knock-in the EGFR C797S mutation in H1975 cells. H1975 cells were cotransfected with CRISPR-EGFP-sgRNA1 and 100-nt donor DNA, which contains the EGFR C797S mutation. CRISPR/Cas9 nuclease-cleaved DNA around the targeted site in the genomic DNA sequence can be repaired by homology-directed repair (HDR) using the provided exogenous homologous donor DNA template. After selection with puromycin and AZD9291, the surviving H1975 cells were seeded in 96-well plates at an average density of one per well by flow cytometry sorting to produce a monoclonal cell population. Sixty-six clones were selected to screen for the knock-in of the EGFR C797S mutation. As shown in Supplementary Figure S1A, 56 clones retained the DNA sequence covering the EGFR C797 region. Each of these clones was then checked for the phenotype of AZD9291 resistance by colony formation assay, for DNA sequence changes, and for the phosphorylation status of EGFR by Western blotting. As shown in Supplementary Figure S1B, 27 clones were resistant to AZD9291 treatment compared to the parental H1975 cells. DNA sequence analysis revealed that the knock-in of EGFR C797S was detected in 19 clones (including 17 homozygous and 2 heterozygous EGFR C797S). However, phosphorylated EGFR was detected in only three clones that were resistant to AZD9291 (Supplementary Figure S1C).

### 2.2. Knock-in of the EGFR C797S Mutation Produces AZD9291-Resistant Phenotypes

**Figure 1:**
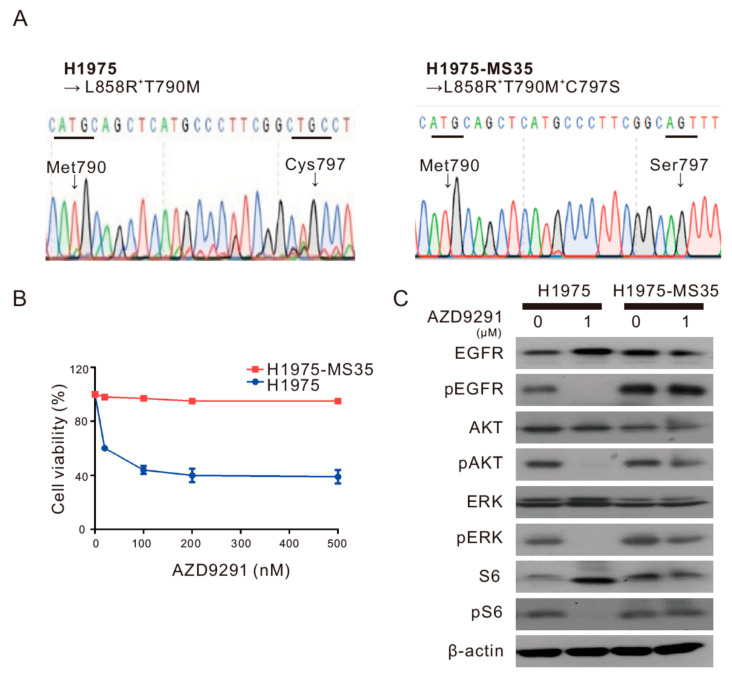
AZD9291-resistant phenotypes of H1975-MS35 cells harboring the knock-in epidermal growth factor receptor (EGFR) C797S mutation. (**A**) Sequencing chromatograms of the EGFR 790–797 region in H1975 cells and H1975-MS35 cells. (**B**) H1975 and H1975-MS35 cells were treated with the indicated concentrations of AZD9291 for 72 h. Cell viability was then assayed by MTT assay. Experiments were carried out in triplicate, and the error bars depict the standard error of the mean. (**C**) H1975 and H1975-MS35 cells were treated with AZD9291 at 1 µM for 24 h, and the cell lysates were assayed for the levels of phospho-EGFR (pEGFR), phospho-AKT (pAKT), phospho-ERK (pERK), phospho-S6 (pS6), EGFR, AKT, ERK, and S6 by Western blotting. β-Actin served as a loading control. The data shown in (**C**) is from one of three similar results.

One clone (H1975-MS35) was confirmed to harbor EGFR L858R, T790M, and C797S triple mutations (Figure 1A) and was used in all of the subsequent studies in this work. As shown in Figure 1B, H1975-MS35 cells were highly resistant to AZD9291 compared to parental H1975 cells. To determine whether AZD9291 resistance was due to the sustained activation of the EGFR pathway by the EGFR C797S mutation, we examined the effect of AZD9291 treatment on the EGFR signaling pathway in H1975-MS35 cells. As shown in Figure 1C, the phosphorylation of EGFR, AKT ERK, and S6 was effectively inhibited by AZD9291 in parental H1975 cells However, the phosphorylation of EGFR and S6 was not inhibited at all in H1975-MS35 cells. We noted that the phosphorylation of AKT and ERK, for unknown reasons, was slightly inhibited by AZD9291 in H1975-MS35 cells. These data indicate that knock-in of the EGFR C797S mutation produces AZD9291-resistant phenotypes.

### 2.3. Exploration of TKI Resistance Mechanisms by Whole-Transcriptome Sequencing and Quantitative Proteomics

To deduce the possible mechanism(s) of EGFR C797S mutation-mediated resistance to TKIs, we conducted whole-transcriptome sequencing and iTRAQ-based quantitative proteomics experiments using H1975 and H1975-MS35 cells. A total of 115 differentially expressed genes (DEGs) were identified by transcriptome analysis (Supplementary Table S1) in H1975-MS35 cells. These DEGs were subjected to ingenuity pathway analysis focusing on the biological networks (Table 1). Such an analysis revealed that the biological networks that are most likely affected by the DEGs are related to myosin heavy chain binding, extracellular matrix structural constituent conferring tensile strength, extracellular matrix structural constituent, myosin binding, actin binding, 1-phosphatidylinositol binding, and cysteine-type endopeptidase activity involved in apoptotic process. A total of 77 differentially expressed proteins (DEPs) were identified by iTRAQ-based quantitative proteomics (Supplementary Table S2). These DEPs were uploaded into STRING (version 11.0) and analyzed for enrichment of categories belonging to biological process pathways in GO (Table 2 and Supplementary Table S3). The analysis revealed that the DEPs are highly correlated with epithelial-mesenchymal transition (EMT), cytoskeletal reorganization, and migratory and invasive properties. The DEG and DEP with the highest score from our whole-transcriptome sequencing and proteomics analyses (Table 1 and Table 2) was AXL, which is involved in myosin heavy chain binding (Table 1), the multicellular organismal process, the cellular response to extracellular stimulus, anatomical structure development, cellular component organization, and cell adhesion (Table 2). AXL is upregulated in mesenchymal NSCLC cell lines as compared to epithelial NSCLC cell lines [18], and these results suggest that H1975-MS35 cells are in a mesenchymal-like cell state.

### 2.4. Upregulation of AXL in NSCLC Cell Lines Carrying EGFR C797S

**Figure 2:**
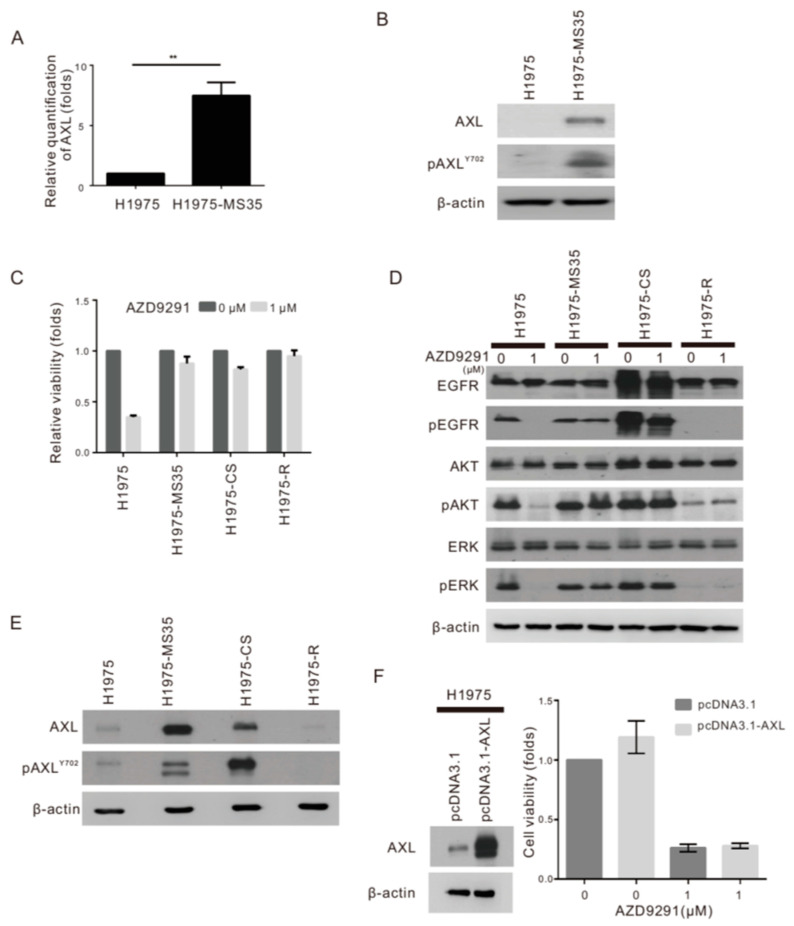
Expression of AXL and sensitivity to AZD9291 in non-small-cell lung cancer (NSCLC) cells. The RNA and protein expression levels of AXL were examined by real-time PCR (**A**) and Western blotting (**B**) in H1975 and H1975-MS35 cells. (**C**) H1975, H1975-MS35, H1975-CS (stable expression of exogenous EGFR-C797S), and H1975-R cells (AZD9291-resistant H1975 cells) were exposed to 1 µM AZD9291 for 72 h and assayed for viability by the MTT method. (**D**) Cells were treated with AZD9291 at 1 µM for 24 h and assayed for the EGFR-dependent pathway by Western blotting. (**E**) Phosphorylated AXL and AXL were detected in H1975, H1975-MS35, H1975-CS, and H1975-R cells by Western blotting. (**F**) Effect of AXL expression on AZD9291 sensitivity. H1975 cells were transfected with AXL or the empty vector (pcDNA3.1) for 24 h, treated with 1 µM AZD9291 for 72 h and analyzed for viability by MTT assay (*right panel*). The expression of AXL in the transfected cells was analyzed by Western blotting of cells cultured for 96 h (*left panel*). The mean ± SD of three independent experiments are shown at the bottom. ** *p* < 0.05 based on Student’s *t*-test. The data shown in (**B**,**D**–**F**) are from one of three similar results.

Since overexpression of AXL is known as an important driver of drug resistance to targeted therapies, immunotherapies, and chemotherapy in various animal models [19,20,21] and has been shown to modulate patients resistant to erlotinib in lung cancer patients [22], we investigated whether AXL overexpression is involved in the TKI resistance associated with acquired EGFR C797S. First, we confirmed that AXL is clearly overexpressed in H1975-MS35 cells compared to H1975 cells by RT-PCR and Western blotting (Figure 2A,B). To address the question of whether the acquisition of the EGFR C797S mutation results in the upregulation of AXL, we examined the effects of ectopic expression of EGFR C797S on the expression of AXL. Similar to the results obtained with H1975-MS35 cells, stable expression of exogenous EGFR C797S in H1975 (H1975-CS) cells resulted in resistance to AZD9291 (Figure 2C), resistance to AZD9291 inhibition of EGFR phosphorylation and downstream signaling (Figure 2D), and upregulation of AXL expression (Figure 2E).

To address the question of whether upregulation of AXL may be associated with acquired resistance to AZD9291 that is independent of EGFR harboring C797S, AZD9291-resistant H1975 cells (H1975-R) were established by continuous exposure of H1975 cells to AZD9291 using a dose-escalation process, and the cells that exhibited at least 100-fold greater IC50 than the parental cells were isolated [10,23]. H1975-R cells were confirmed to harbor only EGFR L858R and T790M mutations (Supplementary Figure S2) and displayed similar resistance to AZD9291 compared to H1975-MS35 and H1975-CS (Figure 2C). However, H1975-R cells did not express activated EGFR and ERK (Figure 2D) and did not upregulate AXL (Figure 2E). Therefore, upregulation of AXL is not an obligatory event associated with the development of resistance to AZD9291. Consistent with this postulate, ectopic expression of AXL in H1975 cells had little or no effect on cell sensitivity to AZD9291 (Figure 2F), indicating that upregulation of AXL alone could not confer resistance to AZD9291.

### 2.5. Effects of AXL Inhibition in NSCLC Cell Lines Carrying EGFR C797S In Vitro

**Figure 3:**
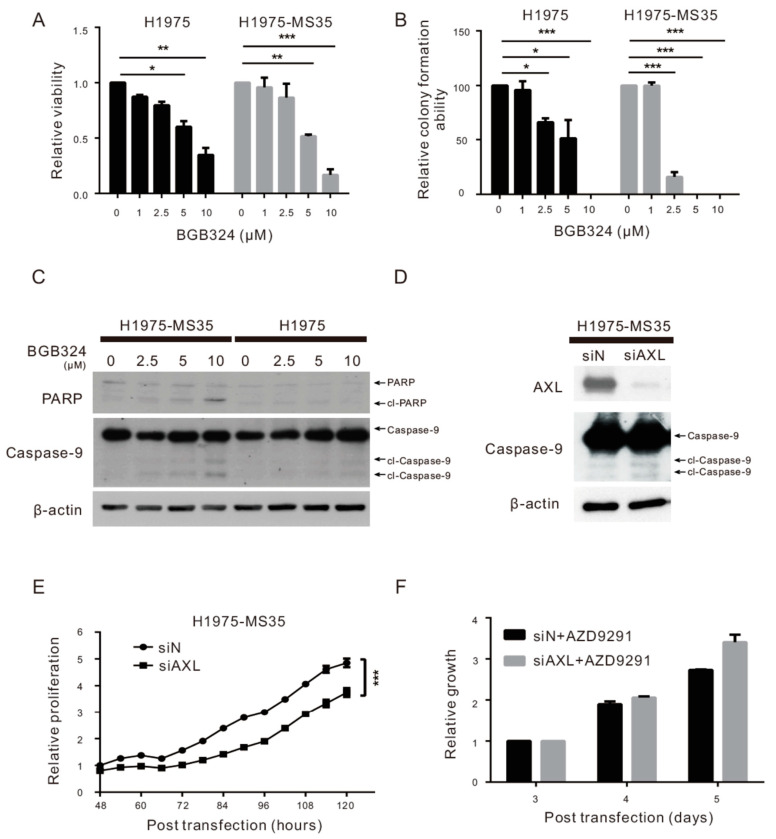
Effects of AXL inhibition in NSCLC cell lines carrying EGFR C797S. (**A**) H1975 and H1975-MS35 cells were treated with various concentrations of BGB324 for 24 h. The viability of treated cells was detected by staining with trypan blue. (**B**) H1975 and H1975-MS35 cells were treated with various concentrations of BGB324 for 24 h and then cultured for an additional eight days in the absence of drugs. The number of foci was scored. (**C**) H1975-MS35 and H1975 cells were treated with BGB324 for 24 h, and the cell lysates were assayed for the cleavage of PARP and caspase-9 by Western blotting. (**D**–**F**) H1975-MS35 cells were transfected with si-N (control) or siAXL and cultured for 48 h. The transfected cells were examined for the expression level of AXL and cleaved caspase-9 by Western blotting (**D**), for cell proliferation by xCELLigence real-time cell analyzer (**E**), and for AZD9291 sensitivity by MTT (**F**). The data shown in A, B, E, and F represent the mean ± SD from three independent experiments; * *p* < 0.05, ** *p* < 0.01, and *** *p* < 0.001 as calculated using Student’s t-test. The data shown in (**C**,**D**) are from one of three similar results.

Although the functional role of AXL in NSCLC remains unclear, the association of AXL upregulation with the acquisition of the EGFR C797S mutation raised the possibility of targeting AXL for the treatment of NSCLC harboring this mutation. Numerous therapeutic agents targeting AXL are under development, including specific and multiple-targeted kinase inhibitors. Specific AXL kinase inhibitors were designed with high selectivity as ATP-competitive inhibitors, including BGB324 and TP-0903 [24]. To explore the therapeutic possibility of AXL inhibition, we used BGB324 (a lead compound of AXL kinase inhibitors in clinical steps) as the AXL kinase inhibitor [25]. In the Supplementary Figure S3, we showed that BGB324 suppressed the AXL phosphorylation in H1975-MS35 cells. Treatment of H1975 and H1975-MS35 cells with BGB324 reduced viability in a dose-dependent manner (Figure 3A). BGB324 appears to exert a greater cytotoxic effect on H1975-MS35 cells than H1975 cells. Similar results were also obtained with the assay assessing clonogenic growth ability (Figure 3B). The greater sensitivity of H1975-MS35 cells to BGB324 is likely attributed to its increased sensitivity to apoptosis induction, as shown by the detection of PARP and caspase-9 cleavage only in H1975-MS35 cells treated with 10 μM BGB324 but not in H1975 cells (Figure 3C). These results indicate that the AXL inhibitor BGB324 has a greater cytotoxic effect on AXL-upregulated H1975-MS35 cells than on control cells.

To address whether the cytotoxic effects of BGB324 were associated with the inhibition of AXL, we examined the effects of AXL downregulation on cell proliferation, apoptosis induction, and resistance to AZD9291. Depletion of AXL slightly increased apoptosis induction (Figure 3D) and reduced cell proliferation (Figure 3E) but had no effects on cell sensitivity to AZD9291 (Figure 3F). These results indicate that AXL inhibition can affect cell proliferation but does not affect cell sensitivity to AZD9291.

### 2.6. Inhibition of AXL Represses Tumor Growth in Xenograft Mice Engrafted with H1975 Cells Harboring the EGFR C797S Mutation

**Figure 4:**
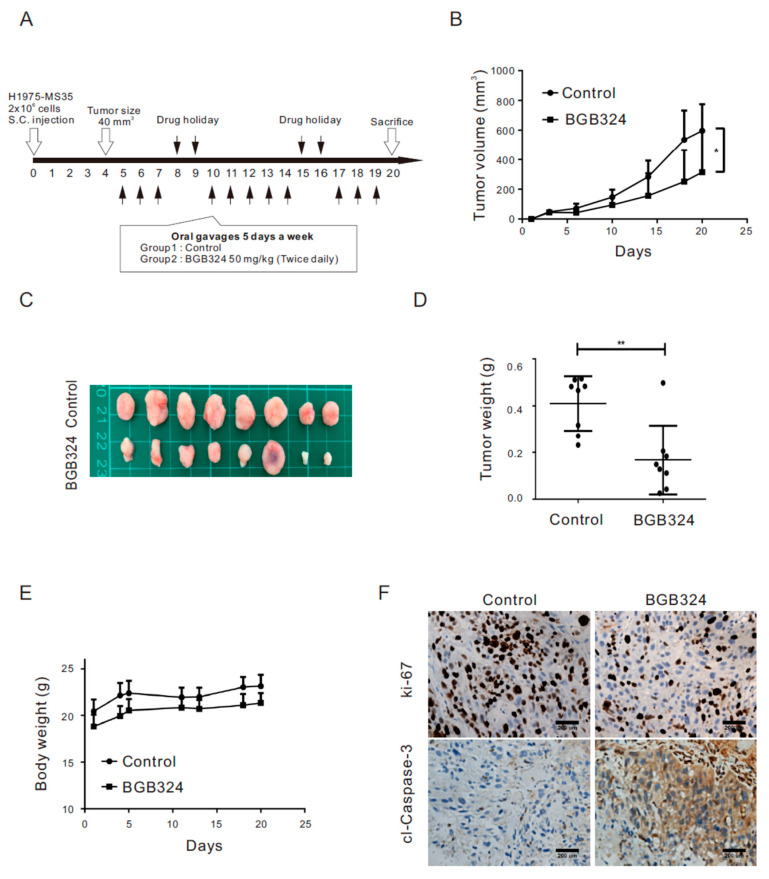
Effect of BGB324 on tumor growth of H1975-MS35 cells in vivo. (**A**) Experimental design for the treatment protocol of H1975-MS35 cells in vivo. H1975-MS35 cells (2 × 10^6^) were inoculated subcutaneously into the right flank of nude mice. Mice were randomly assigned into two groups (n = 8 per group) to receive treatment with BGB324 as shown in the diagram. (**B**) Tumor volume progression. (**C**) Sizes of excised tumors. (**D**) Tumor weights at the end of the study. (**E**) The effect of treatment on the body weights of mice. Data are represented as the mean ± SD of values from eight mice; * *p* < 0.05 and ** *p* < 0.01, as analyzed using Student’s *t*-test. (**F**) IHC staining of Ki-67 and cleaved caspase-3 in the excised tumor sections.

We further evaluated the therapeutic effect of BGB324 in the H1975-MS35 xenograft animal model (Figure 4A). Compared with the control, BGB324 suppressed the growth of H1975-MS35 cell-derived tumors (Figure 4B–D). These treatments did not impact the body weight of mice, suggesting no toxicity (Figure 4E). The suppression of tumor growth by BGB324 appeared to correlate with the suppression of cell proliferation, as assessed by Ki-67, and/or the induction of cell apoptosis, as indicated by cleaved caspase-3 expression (Figure 4F).

## 3. Discussion

In this work, we employed CRISPR/Cas9 editing technology to introduce the EGFR C797S mutation into an NSCLC cell line harboring EGFR L858R/T790M to establish a cellular model for the investigation of the resistance mechanism associated with the acquired C797S mutation and to explore strategies to battle this type of TKI resistance. One of the EGFR C797S knock-in clones (H1975-MS35) was confirmed to harbor EGFR L858R, T790M, and C797S triple mutations (Figure 1A) and was used to investigate the molecular effects of acquired EGFR C797S mutation. As the integrated omics approach is becoming more popular, we employed integrated transcriptome and proteomic analyses to examine the DEGs and DEPs in this work. A total of 115 DEGs were identified by transcriptome analysis (Supplementary Table S1), while a total of 77 DEPs were identified by proteomic analysis (Supplementary Table S2). Eleven upregulated DEGs/DEPs (MMP13, ANXA8, MYH16, COL17A1, SCIN, S100A2, AXL, FAT2, ASNS, SERPINE1, and ITGB4) and three downregulated DEGs/DEPs (CKB, NNMT, and HKDC1) were identified by both transcriptome and proteomic analyses. Among the upregulated DEGs/DEPs, eight candidates (ANXA8, ASNS, AXL, FAT2, ITGB4, MMP13, S100A2, and SERPINE1) are involved with the multicellular organismal process (Table 2 and supplementary Table S3). The DEG and DEP with the highest score from our whole-transcriptome sequencing and proteomics analyses (Table 1 and Table 2) was AXL. We confirmed that AXL is overexpressed in H1975-MS35 cells compared to parental H1975 cells by RT-PCR and Western blotting (Figure 2A,B). The upregulation of AXL appears to be associated with the acquisition of EGFR C797S since stable expression of exogenous EGFR C797S also resulted in upregulation of AXL expression and resistance to AZD9291 in H1975 cells (Figure 2C–E).

AXL activity has been correlated with resistance to targeted therapies. AXL is highly overexpressed in the resistant cells of erlotinib-treated lung cancer, and treatment of an erlotinib-resistant cell line with an AXL inhibitor restored cell sensitivity to erlotinib [22]. AXL overexpression has been reported to induce resistance to the EGFR inhibitor cetuximab in NSCLC and head and neck cancer cell models [26]. The resistance to cetuximab, a monoclonal antibody of EGFR, is thought to be addicted to the EGFR-dependent pathway and transactivation of EGFR and AXL [26]. Therefore, our finding of AXL upregulation in cells harboring EGFR C797S raised the question of whether AXL plays a role in the resistance to AZD9291 in H1975-MS35 cells. However, ectopic expression of AXL in H1975 cells had little or no effect on cell sensitivity to AZD9291 (Figure 2F). Similarly, depletion of AXL in H1975-MS35 cells did not affect cell sensitivity to AZD9291 (Figure 3E). These results indicate that upregulation of AXL alone could not confer resistance to AZD9291 in H1975-MS35 cells.

At present, the mechanism(s) by which EGFR-C797S upregulates AXL expression remain unknown. The transcription factors known to be involved in AXL regulation include HIF-1α, AP1 [27], and stanniocalcin 2 (STC2) [23]. We observed that the expression of HIF-1α was not upregulated in H1975-MS35 and H1975-CS cell lines (data not shown). Moreover, we found that stanniocalcin 1 (STC1) is downregulated in H1975-MS35 cells (Supplementary Table S1). Previous studies have shown that STC1 is regulated by HIF-1 to promote tumor proliferation and metastasis [28]. Therefore, HIF-1α-mediated transactivation is likely not involved in the upregulation of AXL expression in H1975-MS35 cells.

The association of AXL upregulation with the acquisition of the EGFR C797S mutation raised the possibility of targeting AXL for the treatment of NSCLC harboring this mutation. In this work, we have shown that treatment of H1975-MS35 cells with the AXL inhibitor BGB324 reduced the viability of the treated cells and induced apoptosis in vitro (Figure 3). In addition, we showed that BGB324 effectively reduced the growth of H1975-MS35 cell-derived tumors in vivo (Figure 4). However, the molecular mechanism of BGB324 in modulating cell proliferation and death have not been systematically explored. It has been reported that BGB324 induced apoptosis in cancer cells may be dependent [29,30] or independent of AXL inhibition [31]. As BGB324 has been launched into a clinical trial as a second-line treatment for patients who develop resistance after EGFR TKI treatment (NCT02424617), our results suggest that AXL inhibition may be explored as a second-line or potential adjuvant treatment for NSCLC harboring the EGFR C797S mutation.

In summary, we have shown that the knock-in of the EGFR C797S mutation is associated with elevated expression of AXL and that AXL inhibition is effective in slowing the growth of NSCLC cells harboring EGFR C797S. We suggest that AXL inhibition be explored as a second-line or potential adjuvant treatment for NSCLC harboring the EGFR C797S mutation.

## 4. Materials and Methods

### 4.1. Culture Media, Reagents, and Antibodies

Culture media and fetal bovine serum were purchased from Life Technologies (Grand Island, NY, USA). Antibodies were obtained from the following sources: Cell Signaling Technology (Danvers, MA, USA): phospho-AXL (Y702), AXL, phospho-EGFR (Y1068), phospho-AKT (S473), phospho-ERK (T202/Y204), phospho-S6 (S240/244), PARP (# 9541), and S6. Santa Cruz Biotechnology Inc. (Dallas, TX, USA): anti-EGFR (1005), anti-AKT (H-136), anti-ERK (K-23), and horseradish peroxidase (HRP)-conjugated goat anti-mouse IgG, goat anti-rabbit IgG, and donkey anti-goat IgG. AZD9291 and BGB324 were obtained from Selleckchem (Houston, TX, USA).

### 4.2. Cell Lines

The NSCLC cell line H1975 (which harbors EGFR L858R/T790M mutations) and HEK239T cells were purchased from American Type Culture Collection (Manassas, VA, USA). All cell lines were verified by short tandem repeat (STR) analysis. H1975-MS35 is an EGFR C797S knock-in cell line of H1975 as described in this work.

### 4.3. Knock-in of the EGFR C797S Mutation by CRISPR/Cas9 Genome Editing

An all-in-one CRISPR/Cas expression system (a 2-in-1 vector pAll-Cas9.pPuro, National RNAi Core Facility, Taiwan) was employed for the knock-in of EGFR C797S by homology-directed repair (HDR). This pAll-Cas9.pPuro vector expresses Cas9 and guide RNA (gRNA) from the same construct. Four single-guided (sg) RNAs targeting C797 or a nearby region of EGFR were selected and tested for genome editing efficiency by surrogate reporter assay [32] in HEK293T cells. As shown in Supplementary Table S4, EGFR-sgRNA1 showed the highest genome editing efficacy of the target site. We designed a single-stranded (ss) DNA donor template (CCTCCACCGTGCAACTCATCATGCAGCTCATGCCCTTCGGCTCTCTCCTGGACTATGTCCGGGAACACAAAGACAATATTGGCTCCCAGTACCTGCTCA) for precise genome editing. H1975 cells were cotransfected with CRISPR-EGFR-sgRNA1 and (ss) DNA donor template with Lipofectamine 2000 (Life Technology) and then cultured in the presence of puromycin and AZD9291. The surviving cells were plated into 96-well plates at an average density of 1 cell per well using flow cytometry sorting. Genomic DNA from puromycin- and AZD92921-resistant clones was first subjected to the detection of EGFR C797 by PCR and then by Sanger sequencing to detect the presence of edited EGFR C797S as described below.

### 4.4. Detection of EGFR C797S by PCR

The forward primer (CAGCTCATGCCCTTCGGCAGTTTA) is the sequence located at 2628 to 2648 of the EGFR gene, while the reverse primer (GACATCACTCTGGTGGGTATAGATTC) is the sequence located at 2923 to 2948 of the EGFR gene. This pair of primers was used for PCR to amplify a 321-bp product that covers EGFR C797S.

### 4.5. Sanger Sequence Analysis

Genomic DNA was extracted by using the MagNA Pure Compact Nucleic Acid Isolation Kit on the MagNA Pure Compact System (Roche, Pleasanton, CA, USA). The primers used were EGFR-C797-F: CATTCATGCGTCTTCACCTG and EGFR-C797-R: TTATCTCCCCTCCCCGTATC. The target sequences were amplified by a KAPA HiFiHotStart PCR kit (KAPA Biosystems, Pleasanton, CA, USA). The reaction mixtures were run in a 9700 thermal cycler (Applied Biosystems) using the following cycling reactions: 3 min at 95 °C, followed by 25 cycles of 20 s at 95 °C, 20 s at 66 °C, and 30 s at 72 °C, with a final hold at 4 °C. The PCR amplicons were checked by 1.5% agarose gel electrophoresis. The amplicons were purified by using a PCR Fragment Extraction Kit (Geneaid, Taiwan). DNA sequencing was performed using the ABI PRISM BigDye Terminator Cycle Sequencing Ready Reaction Kit v3.1 (Applied Biosystems) and the ABI PRISM 3730XL DNA Analyzer.

### 4.6. Generation of the AZD9291-Resistant H1975-R Cell Line

H1975 cells were seeded into T75 flasks (5 × 10^5^ cells per flask) with growth media. On the following day, AZD9291 was added to the cells at 50 nM (the IC50 concentration for H1975) and cultured with a medium change every 2 days. When the resistant cells were grown to 80% confluency, they were reseeded in media containing twice the concentration of AZD9291. Concentration escalations of AZD9291 were repeated until a final concentration of 3 μM AZD9291 was achieved [10,23]. The resultant AZD9291-resistant cell line was designated H1975-R.

### 4.7. Plasmids, Transfection, and Lentivirus Production and Infection

Lentivirus plasmids that overexpress EGFR T790M and C797S were provided by Dr. Pan-Chyr Yang [33]. Lentiviruses were produced following standard protocols. In brief, HEK293T cells were cotransfected with the indicated lentivirus plasmids and two helper plasmids (pMD.G and pCMVΔR8.91) with Lipofectamine 2000 (Life Technology). Virus-containing medium was collected at day 1, day 2, and day 3 after transfection, and the virus was concentrated by centrifugation. Infection with the indicated lentiviruses was carried out in medium containing 8 μg/mL polybrene. After 24 h, the infected cells were placed in fresh medium and cultured for another 48 h. To generate stably infected cells, cells were cultured in the presence of the selection antibiotic puromycin.

### 4.8. Proteome Analysis with Isobaric Tags for Relative and Absolute Quantitation (iTRAQ)-Based Mass Spectrometry (MS)

The method for proteome profiling of H1975 cells and H1975-MS35 cells is described in detail in the Supplementary Methods. Briefly, proteins from H1975 cells and H1975-MS35 cells were digested with trypsin (Promega, Madison, WI, USA). The resulting peptides were labeled with the iTRAQ™ Reagent Multiplex Kit (AB Sciex, Foster City, CA, USA). We used iTRAQ 114, 115, and 117 for the peptide mixtures from the H1975 control 1, H1975 control 2, and H1975-MS35 groups, respectively. The labeled peptides in each sample were mixed, separated with an online two-dimensional liquid chromatography (LC) system (UltiMate™ 3000 RSLCnano System, Thermo Fisher Scientific, San Jose, CA, USA), and detected by an LTQ-Orbitrap Elite spectrometer (Thermo Fisher Scientific) operating with Xcalibur software (version 2.2 SP1.48, Thermo Fisher Scientific).

Data analysis was performed using Proteome Discoverer software (ver. 1.4.1.14; Thermo Fisher Scientific). The MS/MS spectra were searched against the Swiss-Prot human sequence database (released at 202003, selected for Homo sapiens, 20,198 entries) using the Mascot search engine (version 2.2.0; Matrix Science, London, UK). The peptide confidence setting was set to the following: *p*-value of peptide confidence < 0.01, peptide length > 7 amino acids, and ≥ 2 peptides identified per protein.

For quantification, only proteins with more than 2 quantifiable spectra were accepted. The mean and standard deviation (SD) of the ratios of all proteins for each comparison were obtained. Proteins with ratios larger than the mean plus two SDs (1.6427 and 1.6783 for 117/114 and 117/115, respectively) were defined as upregulated. Proteins with ratios smaller than the mean minus one SD (0.4132 and 0.3898 for 117/114 and 117/115, respectively) were defined as downregulated. Only the proteins differentially displayed in the two sets were considered potential candidates that are differentially expressed in H1975-MS35 cells.

### 4.9. Pathway and Network Analyses

The biological process pathways involving proteins differentially expressed in NSCLC cells with C797S EGFR were revealed by Gene Ontology (GO) database and Search Tool for the Retrieval of Interacting Genes/Proteins (STRING) software (version 11.0, https://string-db.org/cgi/input.pl, Hinxton, UK) analyses, which were performed as previously described [34,35].

### 4.10. Whole-Transcriptome Sequencing

Total RNA was extracted from H1975 and H1975-MS35 cells using TRIzol^®^ Reagent (Invitrogen, Carlsbad, CA, USA). Whole-transcriptome sequencing was performed as described in a previous study [36].

### 4.11. siRNA and Transfection

ON-TARGETplus SMARTpool siRNAs for AXL were purchased from Dharmacon (Dharmacon, Lafayette, CO, USA). The siGENOME nontargeting siRNA pool (Dharmacon) was used as the control. The four siRNAs targeting human AXL mRNA (GenBank accession no. NM_001699.6) were nucleotides 2407–2425 (J-003104-10: ACAGCGAGAUUUAUGACUA), nucleotides 1267–1285 (J-003104-11GGUACCGGCUGGCGUAUCA), nucleotides 2592–2610 (J-003104-12: GACGAAAUCCUCUAUGUCA), and nucleotides 1000–1018 (J-003104-13: GAAGGAGACCCGUUAUGGA). Cells were transfected with siRNA or nontargeting siRNA using Dharmafect 1 transfection reagent (Dharmacon) according to the manufacturer’s instructions. In brief, cells were plated in medium without antibiotics at 40% confluence. After 24 h, cells were transfected with 50 nM siRNA and then incubated for an additional 72 h [35].

### 4.12. Cell Viability Assays

Cell viability assay was carried out by plating 3000 cells/well into 96-well plates and incubated overnight. The cells were drugged with AZD9291 across a five-concentration range from 10 nM to 1000 nM and then incubated for 72 h [37]. Viability was assayed by MTT, trypan blue staining, or by a xCELLigence real-time cell analyzer (Roche Life Science, Pleasanton, CA, USA) according to the manufacturer’s instructions [38].

### 4.13. Assay for Colony Forming Ability

Cells were plated in 6-well plates (500 cells per well) and incubated overnight. Cells were treated with AZD9291 for 72 h. The treated cells were then cultured in fresh medium without AZD9291 for an additional 5 days. Cells were stained with 0.01% crystal violet for 30 min. The plates were dried, and the number of colonies was scored.

### 4.14. Western Blot

Assays for the level of protein expression were performed by Western blotting as previously described [39].

### 4.15. Reverse Transcriptase Polymerase Chain Reaction (RT-PCR)

RNA extraction and RT-PCR were performed as previously described [40].

### 4.16. Xenograft Mouse Tumor Model

The xenograft mouse tumor model was established in six-week-old male Balb/c nude mice (NARLabs, Taipei, Taiwan) by subcutaneously inoculating 2 × 10^6^ human tumor cells (H1975-MS35 cells) in the right flank of the mice. When the implanted tumors reached approximately 40 mm^3^, mice were randomly assigned into two groups of eight mice to receive treatment by oral gavage (vehicle control or 50 mg/kg of BGB324 twice daily). Tumor sizes were measured twice weekly using calipers, and tumor volumes were calculated according to the formula V = 0.5 × W^2^L (W: Width represents small diameter; L: Length represents large diameter). In addition, before each treatment, mice were weighed to monitor signs of drug toxicity twice a week. At the end of the study, mice were sacrificed by CO_2_ asphyxiation, and tumors were excised for histologic analyses.

### 4.17. Immunohistochemistry (IHC)

IHC was performed as described previously [40]. The primary antibodies used for staining targeted cleaved caspase-3 and Ki-67 [40].

### 4.18. Statistical Analysis

Statistical analysis was performed using Student’s *t*-test and was considered significant at *p* < 0.05.

## 5. Conclusions

In this study, we have shown that the knock-in of the EGFR C797S mutation is associated with elevated expression of AXL and that inhibition of AXL is effective in slowing the growth of NSCLC cells harboring EGFR C797S. Our findings suggest that AXL inhibition could be a second-line or a potential adjuvant treatment for NSCLC harboring the EGFR C797S mutation.

## Figures and Tables

**Table 1:** Enrichment analysis of biological processes related to differentially expressed genes in H1975-MS35 cells compared to control H1975 cells by whole-transcriptome sequencing.

Biological Process	*p* Value ^a^	Identified Proteins Involved in the Process
myosin heavy chain binding	0.0000	LIMCH1, CORO1A, AXL
extracellular matrix structural constituent conferring tensile strength	0.0001	COL17A1, COL7A1, COL21A1, COL4A5
extracellular matrix structural constituent	0.0002	COL17A1, COL7A1, COL21A1, HSPG2, TINAGL1, COL4A5
myosin binding	0.0006	LIMCH1, CORO1A, LMTK2, AXL
actin binding	0.0006	SCIN, LIMCH1, EPB41L2, CORO1A, SYNE1, KCNMA1, PKNOX2, DNASE1, ANXA8L1
1-phosphatidylinositol binding	0.0031	SCIN, FRMPD2
cysteine-type endopeptidase activity involved in apoptotic process	0.0031	CASP14,CASP1
endodeoxyribonuclease activity, producing 5’-phosphomonoesters	0.0035	DNASE1L3, DNASE1
actin filament binding	0.0042	SCIN, CORO1A, SYNE1, PKNOX2, ANXA8L1
serine-type peptidase activity	0.0042	F3, DPP6, TMPRSS4, MMP13, KLK6, DPP4
serine hydrolase activity	0.0045	F3, DPP6, TMPRSS4, MMP13, KLK6, DPP4
protease binding	0.0052	TIMP2, SERPINE1, F3, DPP4
3’,5’-cyclic-GMP phosphodiesterase activity	0.0054	PDE1C, PDE2A
virus receptor activity	0.0081	NECTIN1, AXL, DPP4
hijacked molecular function	0.0081	NECTIN1, AXL, DPP4
chloride channel activity	0.0084	FXYD3, SLC26A7, GABRB3
actin monomer binding	0.0098	CORO1A, PKNOX2
anion transmembrane transporter activity	0.0103	FXYD3, SLC6A12, SLC37A2, SLC26A7, GABRB3, SLCO2A1
3’,5’-cyclic-nucleotide phosphodiesterase activity	0.0105	PDE1C, PDE2A
cyclic-nucleotide phosphodiesterase activity	0.0113	PDE1C, PDE2A

^a^ By default, we set the *p*-value cutoff to 0.05 for discovering differentially expressed genes. The *p*-value calculated by cuffdiff with nongrouped sample used “blind mode”, in which all samples were treated as replicates of a single global “condition” and used to build one model for statistic test.

**Table 2:** Enrichment analysis of biological processes related to differentially expressed proteins in H1975-MS35 cells compared to control H1975 cells by iTRAQ-based quantitative proteomics.

Biological Process ^a^	Number of Identified Proteins Involved in the Process	False Discovery Rate
extracellular matrix organization	9	0.0028
cell junction organization	7	0.0074
cell junction assembly	6	0.0075
multicellular organismal process	43 ^b^	0.0087
cellular response to extracellular stimulus	7 ^b^	0.0110
anatomical structure development	36 ^b^	0.0121
anatomical structure formation involved in morphogenesis	12	0.0170
regulation of cellular component movement	12	0.0261
multicellular organism development	33 ^b^	0.0273
response to external stimulus	18 ^b^	0.0273
cellular component organization	35 ^b^	0.0273
cell adhesion	11 ^b^	0.0393
tissue development	16	0.0394
cell-substrate adhesion	5 ^b^	0.0394
regulation of localization	21 ^b^	0.0394
anatomical structure morphogenesis	18 ^b^	0.0398
system development	29 ^b^	0.0406
regulation of locomotion	11	0.0422
positive regulation of cellular component movement	8	0.0422
regulation of cell adhesion	9	0.0427
regulation of cell migration	10	0.0427
regulation of biological quality	26 ^b^	0.0427
positive regulation of locomotion	8	0.0450

^a^ STRING (version 11.0) was applied to functionally annotate enriched proteins, using the annotation category biological process (gene ontology). Processes with at least five protein members and false discovery rate less than 0.05 were considered significant. ^b^ AXL is one of identified proteins involved in the biological process.

## Data Availability

Data is contained within the article or supplementary material.

## Supplementary Materials

The following are available online at https://www.mdpi.com/2072-6694/13/1/111/s1, Figure S1: Screening of the knock-in EGFR C797S clones., Figure S2: Sequencing chromatograms of EGFR T790M and C797 in AZD9291-resistant H1975 (H1975-R) cells., Figure S3, BGB324 suppresses the AXL phosphorylation in H1975-MS35 cells., Table S1: List of genes differentially expressed in the H1975-MS35 cells., Table S2: List of proteins differentially expressed in the H1975-MS35 cells., Table S3: Enrichment analysis of biological processes with differentially expressed proteins in H1975-MS35 cells by proteomics analysis., Table S4: Genome editing efficiency of sg RNAs, Supplementary methods.
