# Supplementary material for: Integrated Omics Analysis of Non-Small-Cell Lung Cancer Cells Harboring the EGFR C797S Mutation Reveals the Potential of AXL as a Novel Therapeutic Target in TKI-Resistant Lung Cancer
Source: Cancers (Basel). 2020 Dec 31;13(1):111. doi: 10.3390/cancers13010111 (PMC7795510; doi:10.3390/cancers13010111)
Supplement: Supplementary file 1 [file cancers-13-00111-s001.zip › cancers-979982-supplementary/3. cancers-979982-supplementary.pdf]

cancers

MDPI

# Supplementary materials: Integrated Omics Analysis of Non-Small-Cell Lung Cancer Cells Harboring the EGFR C797S Mutation Reveals the Potential of AXL as a Novel Therapeutic Target in TKI-Resistant Lung Cancer

**Tong-Hong Wang, Chih-Ching Wu, Kuo-Yen Huang, Yann-Lii Leu, Shuenn-Chen Yang, Ci-Ling Chen and Chi-Yuan Chen**

**Supplementary methods.**

*Tryptic digestion for iTRAQ labeling*

For iTRAQ labeling, proteins of the H1975 and H1975-MS35 cells were reduced with 5 mM tris-(2-carboxyethyl) phosphine hydrochloride (TCEP; Sigma-Aldrich, St. Louis, MO, USA) at 56°C for 1 h and alkylated with 10 mM S-methyl methanethiosulfonate (MMTS; Sigma-Aldrich) at room temperature for 30 min. The protein mixtures were digested with modified, sequencing-grade trypsin (1:10, enzyme:protein; Promega, Madison, WI, USA) at 37°C for 16 h.

The iTRAQ experiments were performed with iTRAQ™ Reagent Multiplex Kit 4-plex (AB Sciex, Forster City, CA, USA) according to the manufacturer's protocol. Briefly, the iTRAQ reagent was reconstituted in ethanol and then mixed with the peptide mixture and incubated with shaking at room temperature for 1.5 h. We used iTRAQ 114, 115, and 117 for the peptide mixtures from the H1975 control 1, H1975 control 2, and H1975-MS35 groups, respectively. The labeled samples were mixed, desalted with a ziptip filled with $C_{18}$ resin (SOURCE™ 5RPC, GE Healthcare, UK), and then dried in a SpeedVac concentrator (Thermo Fisher Scientific). The peptide mixtures were resuspended with SCX-HPLC buffer A containing 0.1% formic acid (FA; Sigma-Aldrich), 30% acetonitrile (ACN; Mallinckrodt Baker, NJ, USA), and 0.5 M $NH_4Cl$ (Sigma-Aldrich).

*Two-dimensional LC-MS for iTRAQ analysis*

The iTRAQ-labeled peptides were separated using an online two-dimensional LC system (UltiMate™ 3000 RSLCnano System, Thermo Fisher Scientific, San Jose, CA, USA). The samples were loaded into a SCX-HPLC column (Luna® 5 μm SCX 100 Å, LC Column 0.5 × 200 mm, Phenomenex, USA) at a constant flow rate of 1 μL/min in buffer A and separated with buffer B (0.1% FA, 30% CAN, and 0.1 M $NH_4Cl$). A linear gradient of buffer B (5% for 2 min, 5-15% for 3 min, 15-35% for 13 min, 35-70% for 25 min, 70-99% for 28 min, 99% for 32 mim) was applied. The resulting peptide fractions were diluted in-line with 0.1% FA prior to trapping onto the column Zorbax 300SB-$C_{18}$ (0.3 × 5 mm, Agilent Technologies, Wilmington, DE, USA). Each fraction was then separated on a homemade column (HydroRP 2.5 μm, 75 μm × 20 cm length) with a 15-μm tip using buffer C (ACN containing 0.1% FA). A linear gradient of buffer C (0-5% for 5 min, 5-28% for 30 min, 28-40% for 10 min, 40-60% for 4 min, 60-90% for 1 min, 90% for 3 min, 90-5% for 2 min, and 5% for 10 min) was applied at a flow rate of 0.3 μL/min.

The LC equipment was connected to the mass spectrometer, LTQ-Orbitrap Elite (Thermo Fisher Scientific), operated by Xcalibur software (version 2.2 SP1.48, Thermo Fisher Scientific). Intact peptides were detected in the Orbitrap at a resolution of 60,000, and the ion source of $(Si(CH_3)_2O)_6H^+$ at *m/z* 445.120025 was used as a lock mass for internal calibration. The 12 data-dependent MS/MS scan events, including 6 collision-induced dissociations (CID) and 6 higher-energy collision-induced dissociations (HCD), were followed by one MS scan for the six most abundant ions in the preview MS scan. The *m/z* values selected for the MS/MS analyses were dynamically excluded for 180 seconds. The electrospray voltage of the source was applied at 1.8 kV.

The microscan with maximum fill times of both MS and MS/MS spectra was 1,000 and 100 ms, respectively. The minimum signal intensity required of MS/MS spectra for both CID and HCD was 10,000, and the normalized collision energy was 35% for CID and 30% for HCD. The *m*/*z* range for MS scan was 350-2,000 Da.

*Protein database searching and iTRAQ data analysis*

Data analysis for the iTRAQ experiments was performed using Proteome Discoverer software (ver. 1.4.1.14; Thermo Fisher Scientific). The MS/MS spectra were searched against the Swiss-Prot human sequence database (released at 202003, selected for Homo sapiens, 20,198 entries) using the Mascot search engine (version 2.2.0; Matrix Science, London, UK). The setting of the MS precursor ion mass was 350-5,000 Da. The search parameters set for CID spectra were to the following: ESI-TRAP instrumentation, precursor mass tolerance 10 ppm, fragment mass tolerance 0.5 Da. The search parameters for HCD spectra were set to the following: ESI-FTICR instrumentation, precursor mass tolerance 10 ppm, fragment mass tolerance 0.05 Da. Peptides with one missed cleavage from the trypsin digestion were allowed. The evaluated modifications included the static methylthio modification on cysteine (+46 Da) and the variable oxidation on methionine (+16 Da) and iTRAQ on the N-terminus and lysine (+144 Da) for both CID and HCD spectra. To ensure a low overall false positive rate for protein identification, the peptide confidence setting was set to the following: $p$-value of peptide confidence $< 0.01$, peptide length $> 7$ amino acids, and $\geq 2$ peptides identified per protein. The identification of the epithelial keratins was excluded to reduce contamination during the experimental process.

The protein quantitative data were exported from Proteome Discoverer. For the protein quantification, only proteins with more than two quantifiable spectra were accepted. The mean and standard deviation (SD) of the ratios of all proteins for each comparison were obtained. Proteins with ratios larger than the mean plus two SD (1.6427 and 1.6783 for 117/114 and 117/115, respectively) are defined as up-regulated. Proteins with ratios smaller than the mean minus one SD (0.4132 and 0.3898 for 117/114 and 117/115, respectively) are defined as down-regulated. Only the proteins differentially displayed in the two sets are considered as potential candidates that are differentially expressed in the H1975-MS35 cells.

**Table S3.** Enrichment analysis of biological processes with differentially expressed proteins in H1975-MS35 cells by proteomics analysis.

| Biological process[a] | Protein count | Identified proteins involved in the biological process | False discovery rate (FDR) |
|---|---|---|---|
| extracellular matrix organization | 9 | COL7A1, ITGA6, ITGB4, ITGB6, LAMA3, MMP13, MMP14, NFKB2, SERPINE1 | 0.0028 |
| cell junction organization | 7 | COL17A1, CSK, FBLIM1, ITGA6, ITGB4, LAMA3, UGT8 | 0.0074 |
| cell junction assembly | 6 | COL17A1, FBLIM1, ITGA6, ITGB4, LAMA3, UGT8 | 0.0075 |
| multicellular organismal process | 43 | ABAT, AKR1C1, ALDH1A3, ANXA8, ARHGDIB, ASNS, **AXL**, CKB, COL7A1, CRABP2, CSK, CSRP2, CTSH, DSC2, EPHB4, ERAP1, FAT2, GJC3, GSTM3, HCCS, IGF1R, IRF6, ITGA6, ITGB4, ITGB6, LAMA3, MMP13, MMP14, NFKB2, NNMT, OPA1, PAIP2, PIR, PLAU, PRDX5, PROCR, S100A2, SEMA3C, SERPINE1, SLC38A2, SORL1, TUBB2B, UGT8 | 0.0087 |
| cellular response to extracellular stimulus | 7 | ASNS, **AXL**, DSC2, GLUL, ITGA6, OPA1, SLC38A2 | 0.0110 |
| anatomical structure development | 36 | ABAT, AKR1C1, ALDH1A3, ARHGDIB, ASNS, **AXL**, CKB, COL17A1, COL7A1, CPT1A, CRABP2, CSK, CSRP2, CTSH, DSC2, EPHB4, ERAP1, GJC3, GSTM3, HCCS, IRF6, ITGA6, ITGB4, LAMA3, MMP13, MMP14, NFKB2, NNMT, OPA1, PIR, SEMA3C, SERPINE1, SLC38A2, SORL1, TUBB2B, UGT8 | 0.0121 |
| anatomical structure formation involved in morphogenesis | 12 | ALDH1A3, COL7A1, EPHB4, ERAP1, ITGA6, ITGB4, LAMA3, MMP14, NFKB2, SEMA3C, SERPINE1, UGT8 | 0.0170 |
| regulation of cellular component movement | 12 | ARHGDIB, CTSH, DSC2, IGF1R, ITGA6, LAMA3, MMP14, PLAU, SEMA3C, SERPINE1, SORL1, TUBB2B | 0.0261 |
| multicellular organism development | 33 | ABAT, ALDH1A3, ARHGDIB, ASNS, **AXL**, CKB, COL7A1, CRABP2, CSK, CSRP2, CTSH, DSC2, EPHB4, ERAP1, GJC3, GSTM3, HCCS, IRF6, ITGA6, ITGB4, LAMA3, MMP13, MMP14, NFKB2, NNMT, OPA1, PIR, SEMA3C, SERPINE1, SLC38A2, SORL1, TUBB2B, UGT8 | 0.0273 |
| response to external stimulus | 18 | ALDH1A3, APOBEC3B, ASNS, **AXL**, BAIAP2L1, DSC2, ERAP1, GLUL, HLA-E, ITGA6, MMP14, NFKB2, OASL, OPA1, PLAU, SEMA3C, SERPINE1, SLC38A2 | 0.0273 |
| cellular component organization | 35 | AKR1C1, ALDH1A3, ANXA8, **AXL**, BAIAP2L1, COL17A1, COL7A1, CPT1A, CRABP2, CSK, FBLIM1, GLUL, HCCS, HIST1H1D, HMGN5, IGF1R, ITGA6, ITGB4, ITGB6, KCTD14, LAMA3, MINA, MMP13, MMP14, NFKB2, OPA1, PADI3, PDLIM4, RHOF, SCIN, SEMA3C, SERPINE1, SYTL1, | 0.0273 |

| | | | |
|---|---|---|---|
| | | TUBB2B, UGT8 | |
| cell adhesion | 11 | **AXL**, COL17A1, COL7A1, DSC2, EPHB4, FAT2, FBLIM1, ITGA6, ITGB4, ITGB6, LAMA3 | 0.0393 |
| tissue development | 16 | AKR1C1, ALDH1A3, COL17A1, COL7A1, CPT1A, CRABP2, CTSH, DSC2, GSTM3, IRF6, ITGA6, ITGB4, LAMA3, MMP13, MMP14, SEMA3C | 0.0394 |
| cell-substrate adhesion | 5 | **AXL**, COL17A1, ITGA6, ITGB4, ITGB6 | 0.0394 |
| regulation of localization | 21 | ABAT, ARHGDIB, **AXL**, CPT1A, CSK, CTSH, DSC2, GLUL, HLA-E, IGF1R, ITGA6, LAMA3, MMP14, OPA1, PLAU, SCIN, SEMA3C, SERPINE1, SORL1, SYTL1, TUBB2B | 0.0394 |
| anatomical structure morphogenesis | 18 | ALDH1A3, **AXL**, COL7A1, CRABP2, CTSH, EPHB4, ERAP1, HCCS, ITGA6, ITGB4, LAMA3, MMP13, MMP14, NFKB2, OPA1, SEMA3C, SERPINE1, UGT8 | 0.0398 |
| system development | 29 | ABAT, ALDH1A3, ASNS, **AXL,** CKB, CRABP2, CSK, CTSH, DSC2, EPHB4, ERAP1, GJC3, GSTM3, HCCS, IRF6, ITGA6, ITGB4, MMP13, MMP14, NFKB2, NNMT, OPA1, PIR, SEMA3C, SERPINE1, SLC38A2, SORL1, TUBB2B, UGT8 | 0.0406 |
| regulation of locomotion | 11 | ARHGDIB, CTSH, IGF1R, ITGA6, LAMA3, MMP14, PLAU, SEMA3C, SERPINE1, SORL1, TUBB2B | 0.0422 |
| positive regulation of cellular component movement | 8 | CTSH, IGF1R, ITGA6, MMP14, PLAU, SEMA3C, SERPINE1, TUBB2B | 0.0422 |
| regulation of cell adhesion | 9 | ABAT, ARHGDIB, CSK, HLA-E, ITGA6, LAMA3, MMP14, PLAU, SERPINE1 | 0.0427 |
| regulation of cell migration | 10 | ARHGDIB, CTSH, IGF1R, ITGA6, LAMA3, MMP14, PLAU, SEMA3C, SERPINE1, SORL1 | 0.0427 |
| regulation of biological quality | 26 | ABAT, AKR1C1, ALDH1A3, ANXA8, ATP13A3, **AXL**, BAIAP2L1, CKB, CPT1A, CRABP2, CSK, CTSH, DSC2, ERAP1, FBLIM1, GLUL, HKDC1, OPA1, PAIP2, PLAU, PRDX5, PROCR, SCIN, SERPINE1, SORL1, SYTL1 | 0.0427 |
| positive regulation of locomotion | 8 | CTSH, IGF1R, ITGA6, MMP14, PLAU, SEMA3C, SERPINE1, TUBB2B | 0.0450 |

[a] STRING (version 11.0) was applied to functionally annotate enriched proteins, using the annotation category biological process (gene ontology). Processes with at least five protein members and false discovery rate less than 0.05 were considered significant.

**Table S4. Genome editing efficiency of sg RNAs.** To examine the efficiency of four sgRNA-targeting sequence cleaved by CRISPR system, the sgRNA was cloned into the dual-reporter surrogate plasmid between EGFP and mCherry genes. The surrogate reporter plasmid was transfected into HEK293T cells by Lipofectamine 2000. After 24 hours, transfected cells were examined for the expression of mCherry fluorescence to determine the efficiency of genome editing.

| ID | Sequence | Status | Efficiency |
|---|---|---|---|
| EGFR sgRNA1 | GTTCCCGGACATAGTCCAGG | OK | 68% |
| EGFR sgRNA2 | GCTGCCTCCTGGACTATGTC | OK | 45% |
| EGFR sgRNA3 | GGTGTTCCCGGACATAGTCC | OK | 58% |
| EGFR sgRNA4 | GTCATGCAGCTCATGCCCTT | OK | 63% |
| EGFR surrogate | atcacgcagctcatgcccttcggctgcctcctggactatgtccgggaacaca | OK | |

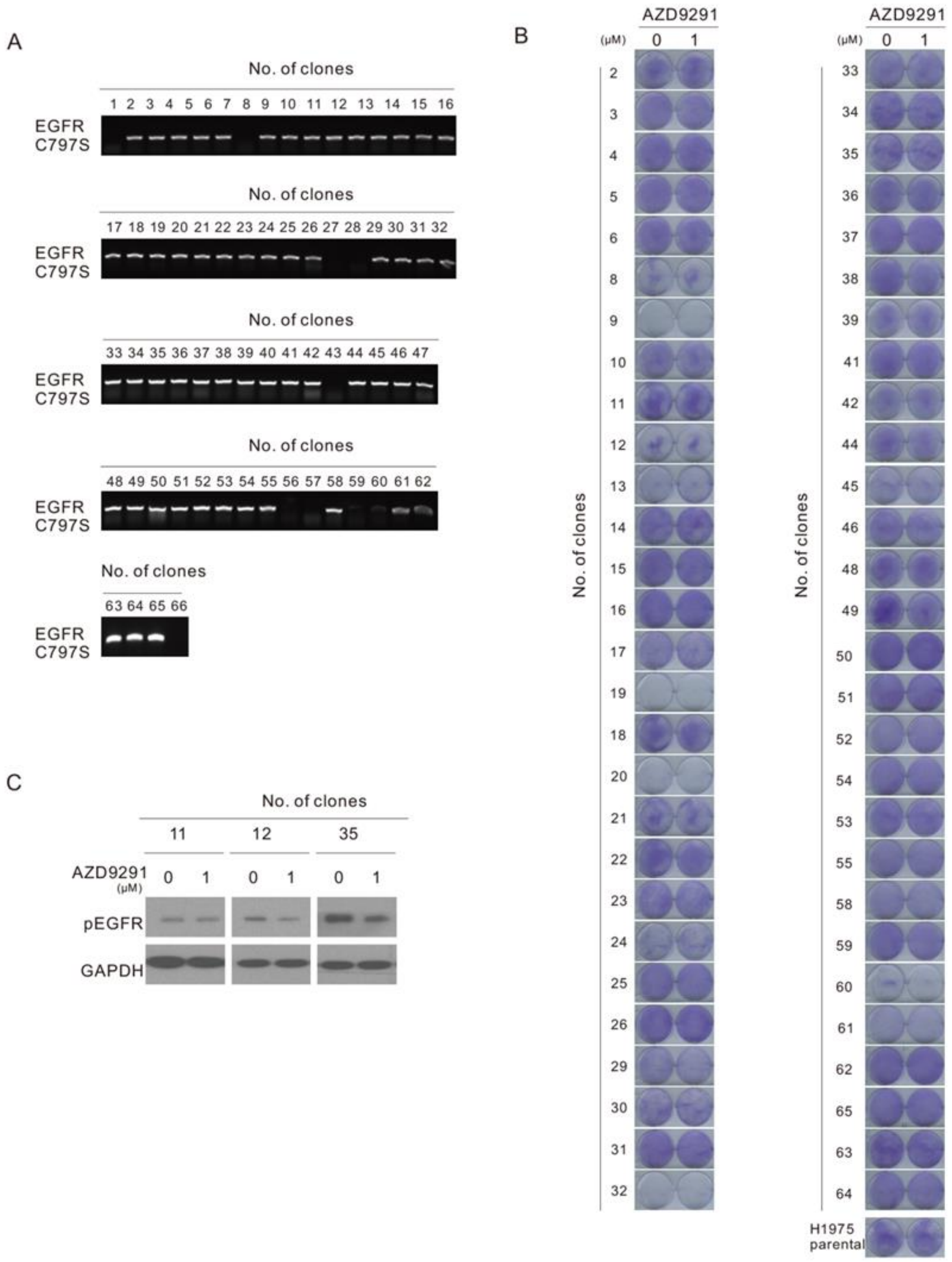


**Figure S1.** Screening of the knock-in EGFR C797S clones. (A) Detection of EGFR C797 by PCR. Sixty-six clones were selected to screen for the knock-in of the EGFR C797S mutation. The detection of the 321-bp PCR product indicates that the clone retained the DNA sequence covering the EGFR C797 region. (B) Fifty-six clones that retained the EGFR C797 sequence were tested for AZD9291 sensitivity by colony formation assay. (C) The phosphorylation status of EGFR was assayed by Western blotting. Phosphorylated EGFR was detected only in 3 clones.

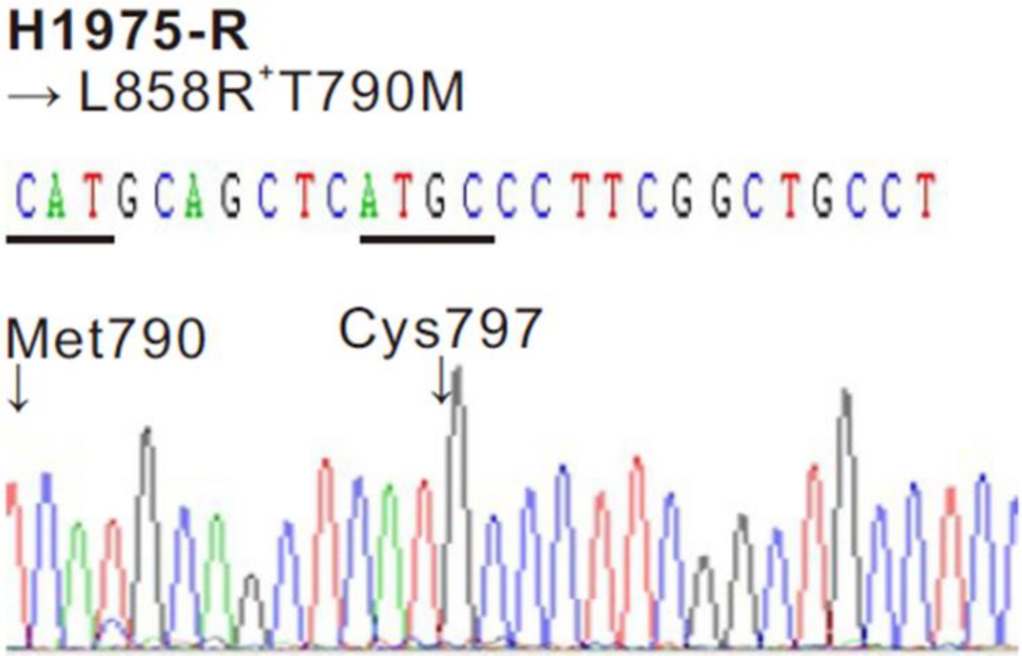


**Figure 2.** Sequencing chromatograms of EGFR T790M and C797 in AZD9291-resistant H1975 (H1975-R) cells.

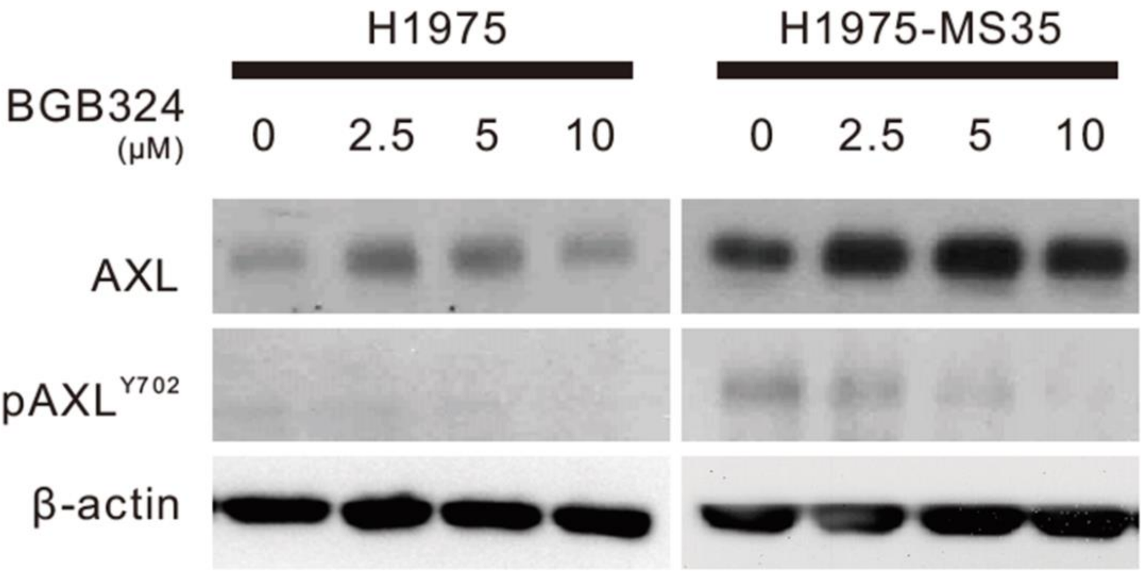


**Figure 3.** Effects of AXL inhibition in NSCLC cell lines carrying EGFR C797S. H1975-MS35 and H1975 cells were treated with BGB324 for 24 h, and the cell lysates were assayed for phosphorylated AXL and AXL by Western blotting.
