# Supplementary material for: Integrated Omics Analysis of Non-Small-Cell Lung Cancer Cells Harboring the EGFR C797S Mutation Reveals the Potential of AXL as a Novel Therapeutic Target in TKI-Resistant Lung Cancer
Source: Cancers (Basel). 2020 Dec 31;13(1):111. doi: 10.3390/cancers13010111 (PMC7795510; doi:10.3390/cancers13010111)

Figure 2

C.

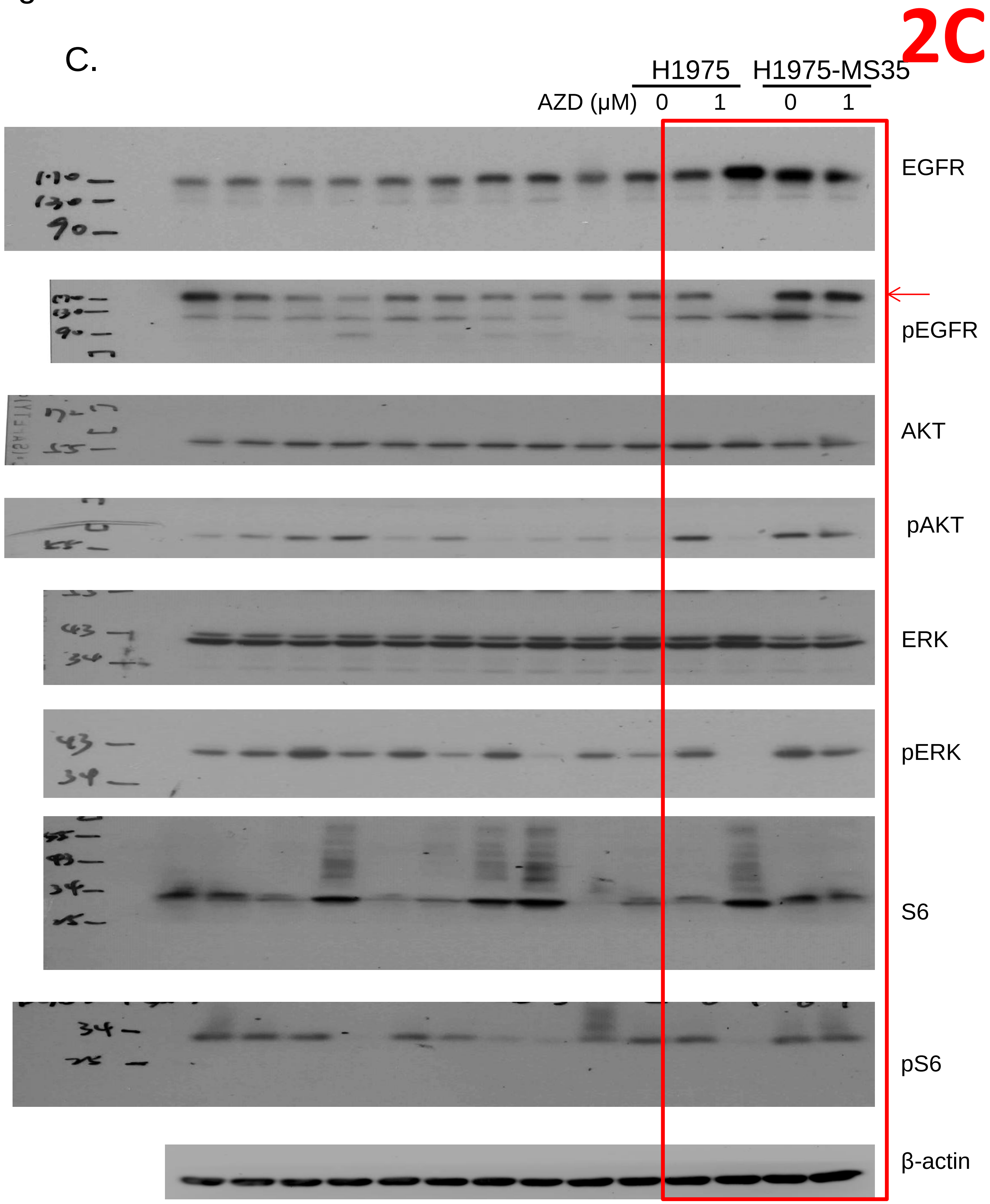
2C
H1975
H1975-MS35
AZD (μM) 0 1 0 1
EGFR
pEGFR
AKT
pAKT
ERK
pERK
S6
pS6
β-actin

Figure 3

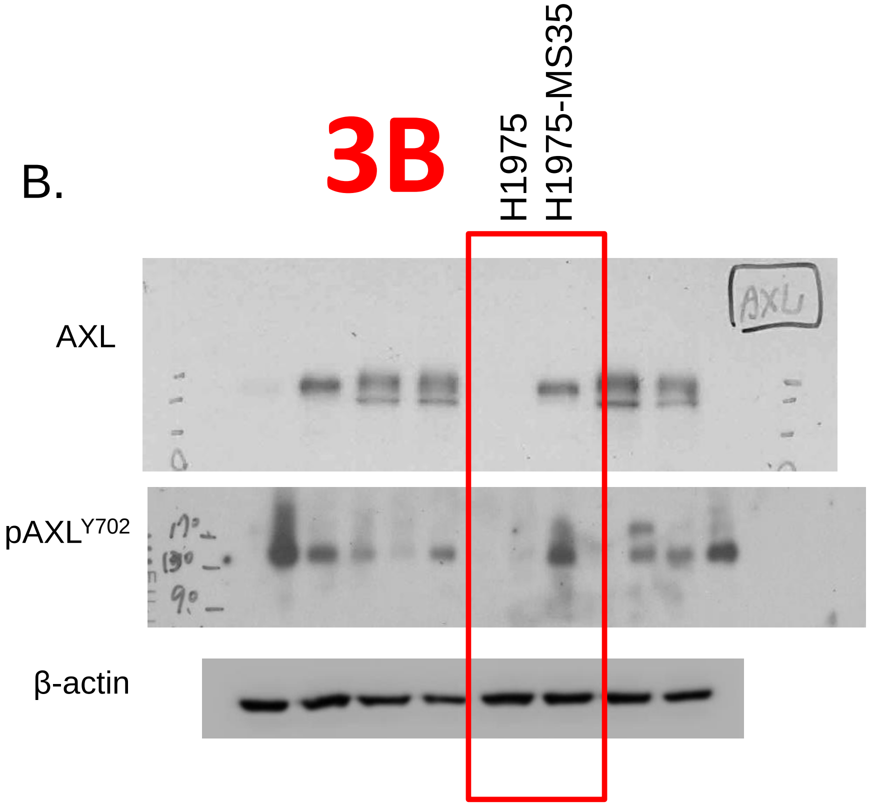
3B
B.
H1975
H1975-MS35
AXL
pAXLY702
β-actin

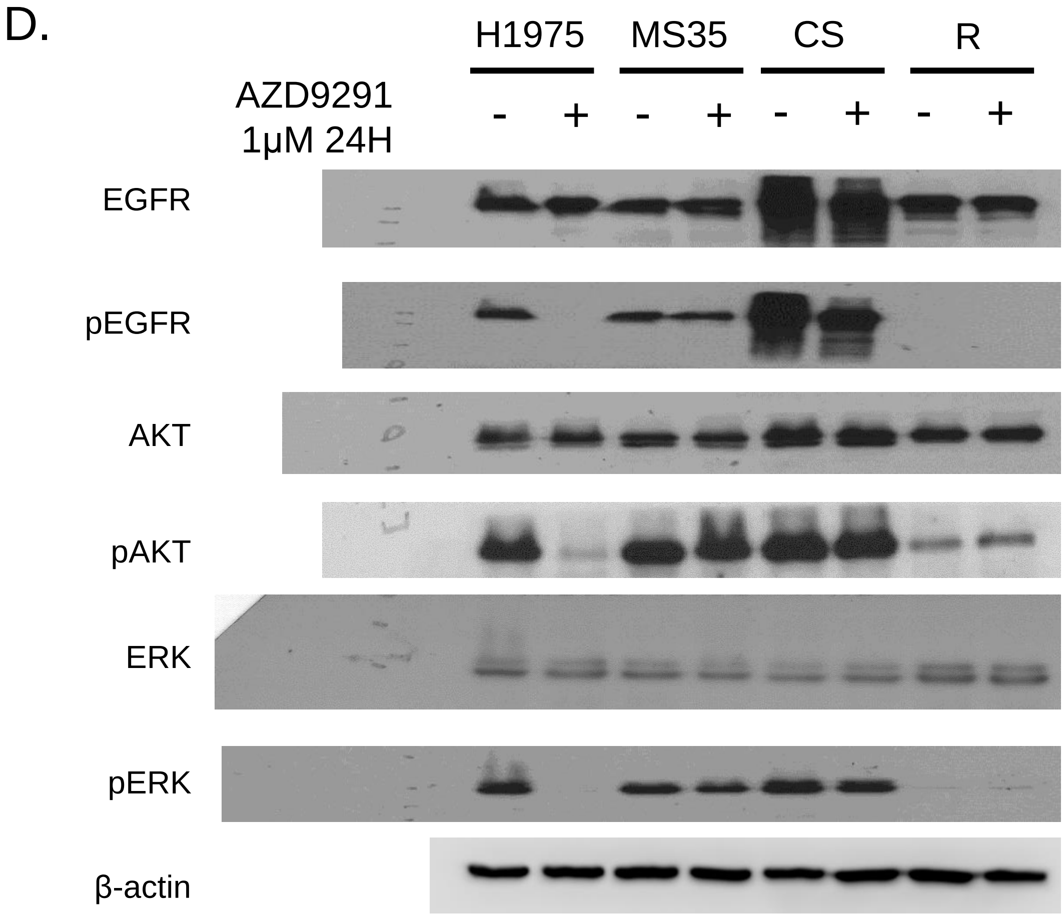
D.
H1975
MS35
CS
R
AZD9291
1μM 24H
- + - + - + - +
EGFR
pEGFR
AKT
pAKT
ERK
pERK
β-actin

Figure 3

E.

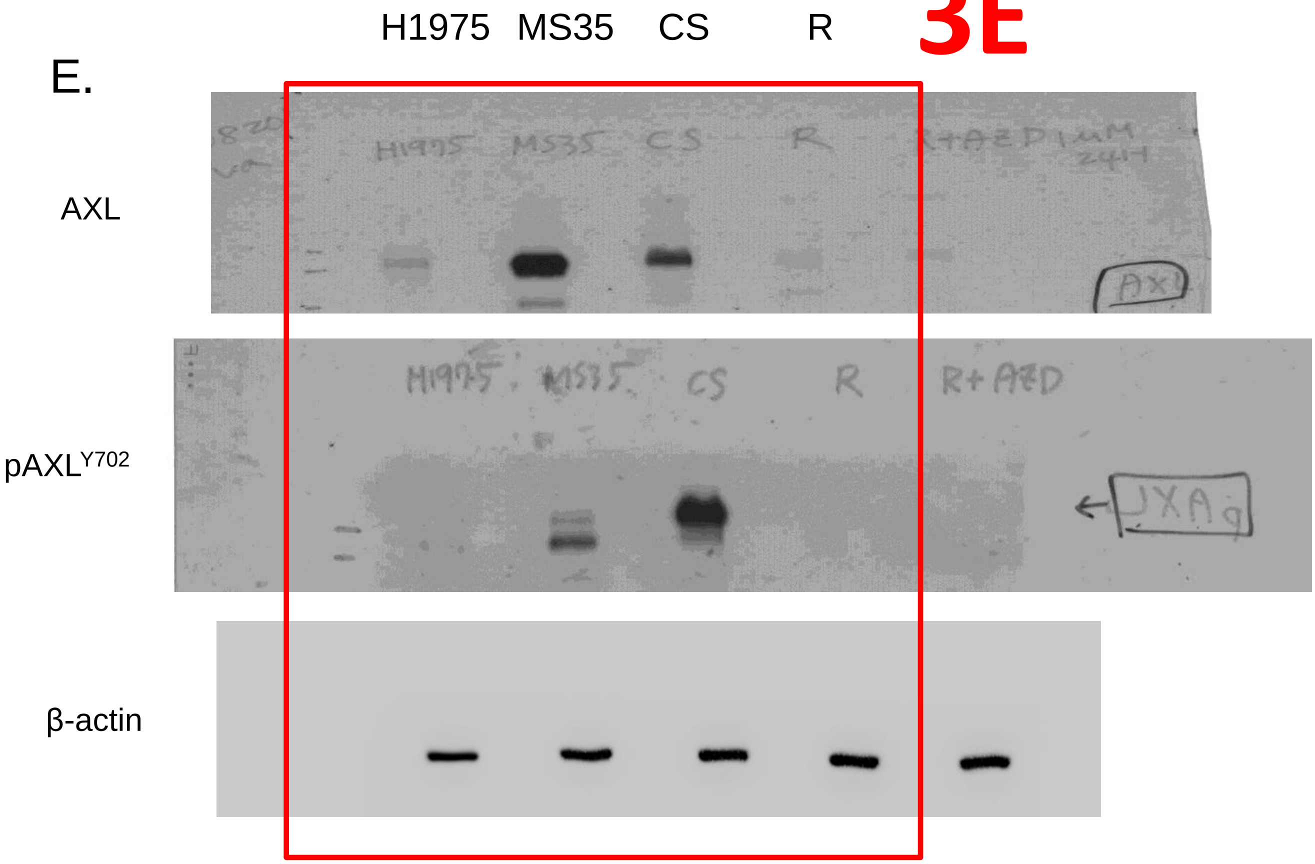

F.

H1975

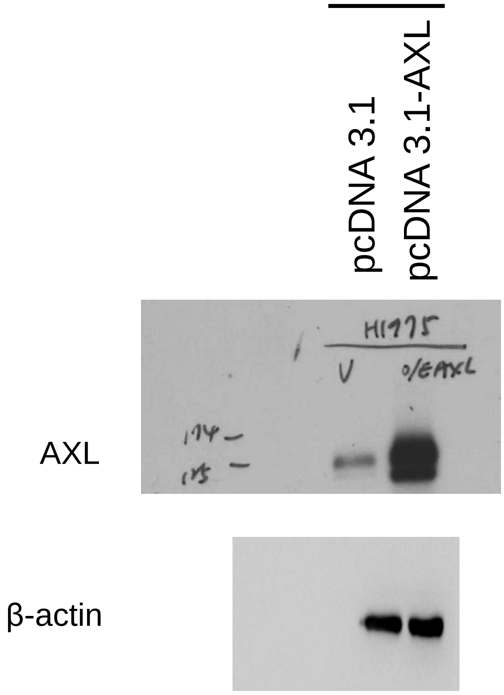

Figure 4

C

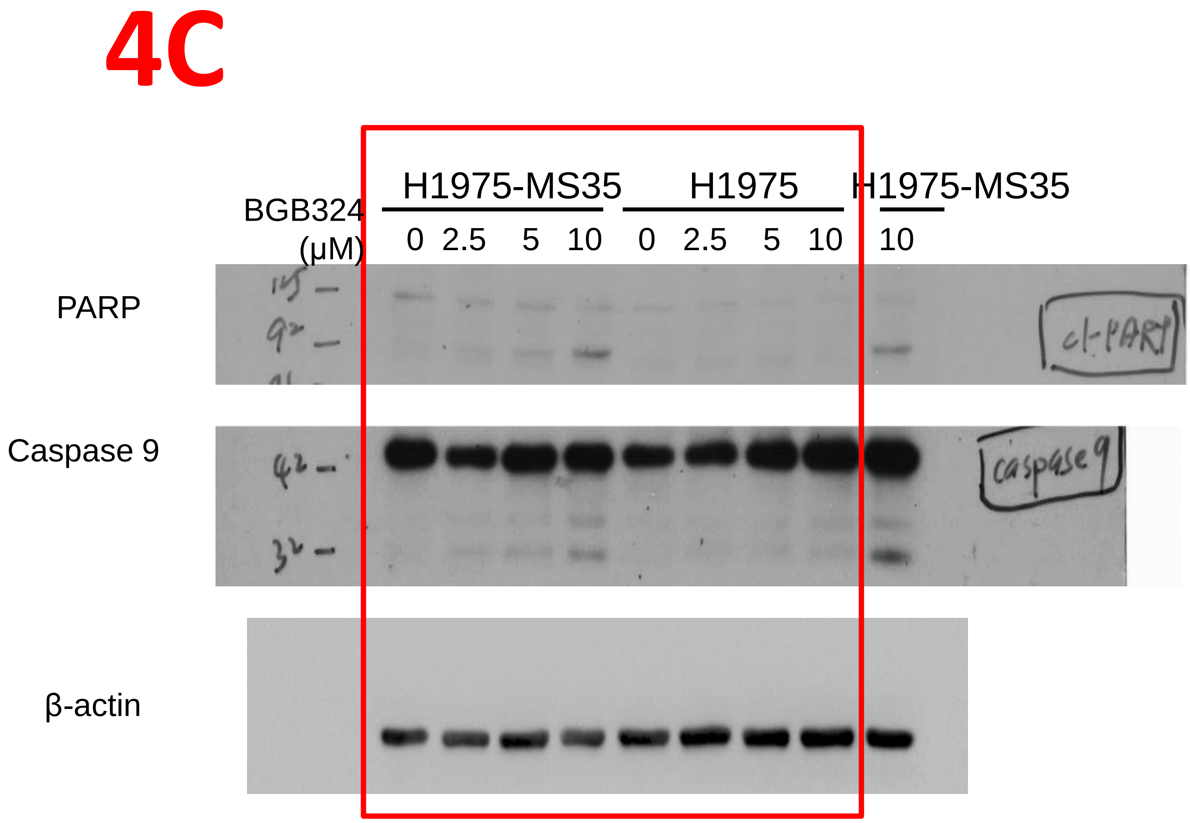
4C
H1975-MS35
H1975
H1975-MS35
BGB324 (μM)
0 2.5 5 10 0 2.5 5 10 10
PARP
Caspase 9
β-actin
cl-PARP
caspase 9

D

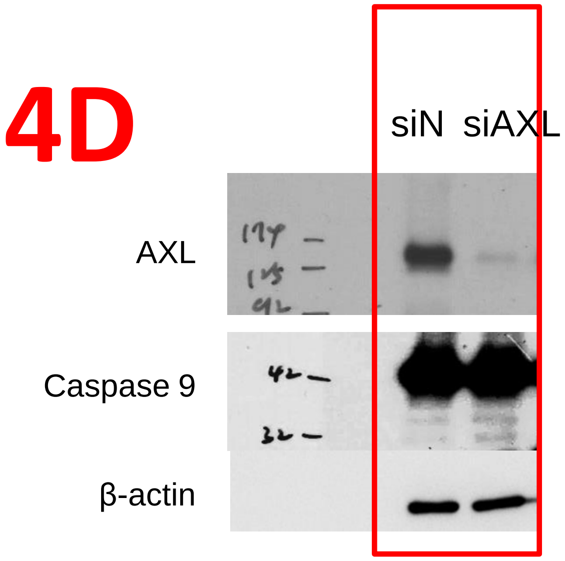
4D
siN siAXL
AXL
Caspase 9
β-actin

Supplementary Figure 1

C

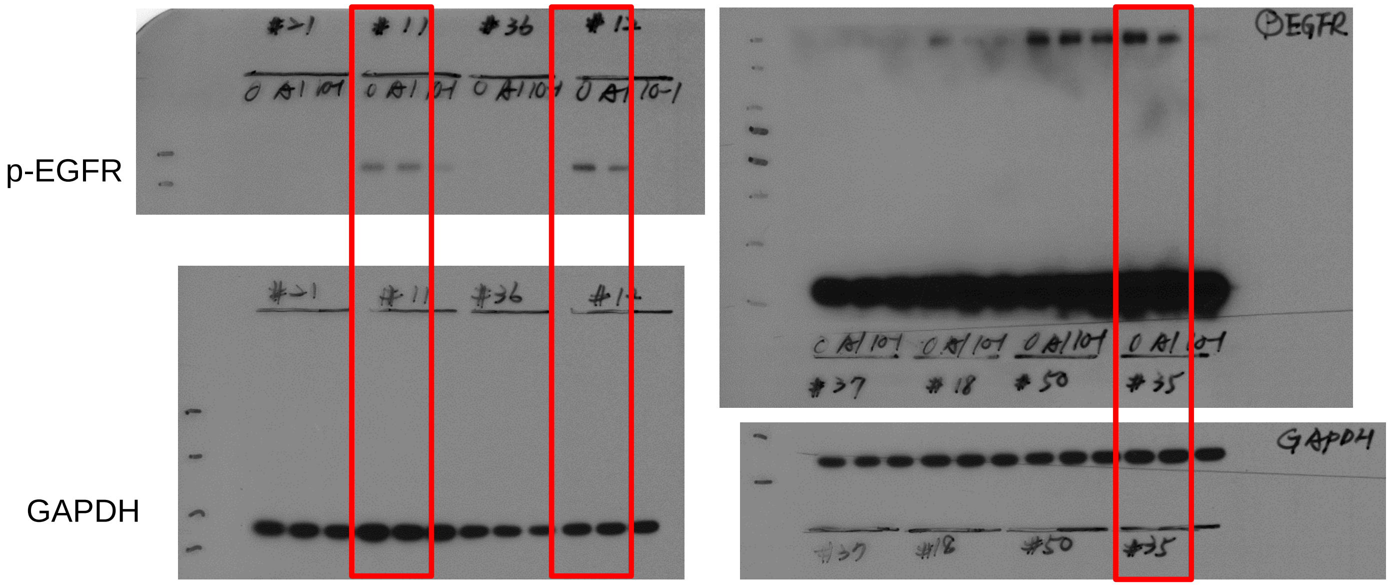

Supplement: Supplementary file 1 [file cancers-13-00111-s001.zip › cancers-979982-supplementary/cancers-979982-original-images.pdf]
